# *Glycyrrhiza uralensis*-*Atractylodes macrocephala* combination supplementation enhances broiler growth performance, immunity, and intestinal health, potentially mediated through gut microbiota and metabolites

**DOI:** 10.3389/fvets.2026.1855302

**Published:** 2026-05-28

**Authors:** Jiamin Tian, Guohua Zhang, Naifei He, Liyou Tan, Jiahui Wu, Yan Chen, Liping Yao, Yushan Bi, Yingpai Zhaxi, Jianxiong Lu, Susu Jiang

**Affiliations:** School of Life Sciences and Engineering, Northwest Minzu University, Lanzhou, China

**Keywords:** broiler, *Glycyrrhiza uralensis*-*Atractylodes macrocephala* combination, growth performance, gut metabolite, gut microbiota, immune function

## Abstract

The poultry industry requires effective antibiotic alternatives to enhance growth and maintain health. This study investigated the effects of the *Glycyrrhiza uralensis*-*Atractylodes macrocephala* combination (GA) on growth, meat quality, and health in broilers. One-day-old male *Lingnan* yellow broilers were randomly assigned to a control group (fed a basal diet) and two treatment groups that received diets supplemented with 0.1% (LGA) and 0.3% (HGA) GA, respectively. The experiment lasted for 84 days. The results showed that HGA significantly enhanced average daily gain (ADG) in broilers aged 29–56, 57–84, and 1–84 days, accompanied by a decreased feed-to-gain ratio (F/G). HGA increased carcass performance by increasing leg muscle rate and decreasing abdominal fat rate at day 84, and improved meat quality by reducing L* and b* values and shear force, and increasing a* value in breast muscle. HGA elevated the thymus and bursa of Fabricius indices and serum immunoglobulin A (IgA), immunoglobulin M (IgM), and interleukin-10 (IL-10) levels, while decreasing interleukin-2 (IL-2) and tumor necrosis factor-α (TNF-α) levels. Moreover, HGA increased jejunal villus height (VH) and villus height/crypt depth (VH/CD), and secretory immunoglobulin A (sIgA) content and mRNA expression of *ZO-1* and *CLDN1*. It also modulated the cecal microbiota composition and altered microbial interactions in an age-dependent way. Specifically, HGA increased the abundance of short-chain fatty acids-producing or anti-inflammatory-associated bacteria, such as *Rikenellaceae_RC9_gut_group*, *Negativibacillus*, *Enterococcus*, *Butyricicoccus*, and *Muribaculaceae*, while decreasing the abundance of pro-inflammatory-associated bacteria, such as *Parasutterella*, *Desulfovibrio*, and *Campylobacter*. Metabolomic analysis revealed that HGA altered cecal metabolic profiles by upregulating key metabolites, including 3α, 7α, 12α-trihydroxy-5β-cholestanoate, enoxolone, and butyric acid. In conclusion, HGA enhances production performance, immune function, and intestinal barrier integrity in broilers, where shifts in gut microbiota and metabolites may contribute to these beneficial outcomes. These findings provide a solid scientific basis for the design and utilization of herbal feed additives.

## 1 Introduction

Under intensive modern production system, broilers are susceptible to various stress factors, such as diseases and nutritional and environmental challenges, which often impair performance and health by exacerbating stress responses and reducing immunity (1). Natural feed additives including medicinal plant extracts and probiotics, offer a solution by enhancing productivity while maintaining health. Certain plant-derived additives may serve as a natural and multifunctional alternatives to antibiotic additives, offering the potential to mitigate disease risk while minimizing concerns regarding the development of antimicrobial resistance (2).

The traditional Chinese medicine (TCM) consists of numerous herbs with diverse pharmacological effects and is often used in formulas to achieve synergistic effects or mitigate potential adverse reactions (3). Consequently, the primary function of a formula may vary based on its composition. Both *Glycyrrhiza uralensis* Fisch. (GU) and *Atractylodes macrocephala* Koidz. (AM) are traditional medicinal and edible plants with a long history of dietary and medicinal application, with their usable parts being the root and rhizome (4, 5). As two of the most widely utilized herbs in TCM, AM and GU are prominently featured in the Treatise on Cold Damage (“Shang Han Lun”, a foundational TCM text), and constitute the core ingredients in multiple classical formulas, such as *Sijunzi Decoction* and *Shenling Baizhu* (*Atractylodes macrocephala*) *Powder*. These formulae exhibit therapeutic effects such as spleen-strengthening, dampness-dispelling, and fluid retention-resolving activities (6).

AM is a perennial herbaceous plant in the *Asteraceae* family that has numerous pharmacological effects, including enhancing gastrointestinal function, lowering blood glucose and lipids, and boosting immunity and exerting anti-inflammatory effects. Its primary bioactive constituents include atractylone, which has antioxidant and anti-inflammatory properties; atractylenolide I, which aids digestion and also possesses anti-inflammatory effects (7); and polysaccharides that enhance immunity (8). Five sesquiterpenes in AM have an inhibitory effect on acute inflammation (9). GU is commonly used in TCM to relieve pain, strengthen the spleen and stomach, and function as an expectorant and cough suppressant (10). The main active components in GU are glycyrrhiza polysaccharides, flavonoids, and triterpene saponins (such as glycyrrhizic acid and glycyrrhizinic acid) (11). GU extract demonstrates a wide range of pharmacological activities, including anti-inflammatory, antioxidant, antiviral, and immunoregulatory effects (12). We previously established that GU effectively alleviated the damage to intestinal health caused by deoxynivalenol (DON) and zearalenone (ZEN) contamination (13).

Herbal formulas are the primary clinical treatments used in TCM. Accumulating evidence indicates that monotherapy with a single active pharmaceutical ingredient or a single herbal preparation frequently demonstrates limited therapeutic efficacy, attributable to the multifactorial complexity of physiological regulation and disease pathogenesis (14). The fundamental principle governing TCM formulations is the principle of herbal compatibility, which involves combining two or more compatible herbs to achieve greater efficacy than individual herbs alone (15). TCM formulas have demonstrated efficacy in treating complex diseases and their symptoms through their ability to target multiple mechanisms of action (16). GU exerts pharmacological synergy and modulates herb compatibility in traditional formulations (17). Studies have shown the synergistic effects and compatibility between GU and AM. AM enhanced the spleen-strengthening effect of GU, whereas GU potentiated AM’s spleen-tonifying activity by alleviating its drying property (18). Kim (19) demonstrated that AM increased GU bioavailability, prolonged its elimination half-life, and enhanced its therapeutic efficacy. GU countered AM-induced upregulation of tumor protein p53 (*Trp53*) and cyclin-dependent kinase inhibitor 1A (*p21*) genes in mouse intestinal epithelial cells and promoted intestinal cell proliferation (20). Leveraging the distinct biological functions and potential synergistic effects of GU and AM, we developed a TCM additive designated as GA, which is formulated using extracts derived from both the herbs. While individual active components of both GU and AM, such as polysaccharides and flavonoids, have been studied as feed additives for their effects on animal growth and health (21–23), extracts containing a spectrum of active components are more practical for production applications due to their multi-target biological activities and simplified manufacturing processes. However, it remains unclear whether GA extracts as feed additives can exert beneficial effects on the production and health of broiler chickens. This study investigated the effects of dietary GA supplementation on the production performance, immunity, and intestinal health of *Lingnan yellow* broiler chickens, a medium-growing strain renowned for its excellent meat quality. Additionally, an analysis of the cecal microbiome and metabolome will clarify the underlying mechanisms, offering actionable insights for developing herbal-based alternatives to antibiotic additives in poultry.

## 2 Materials and methods

### 2.1 Animals, diets, and experimental design

The *Glycyrrhiza uralensis* extract (GUE; prepared from the root of GU) and *Atractylodes macrocephala* extract (AME; prepared from the rhizome of AM) used in the present study were supplied by Yalan Pharmaceutical Co. (Gansu, China). Among them, the content of glycyrrhizic acid and glycyrrhizin in GUE was 7 and 0.5%; the content of atractylone and atractylenolide in AME were 0.3 and 0.05%. GUE and AME were mixed in a 1:1 ratio to prepare the *Glycyrrhiza-Atractylodes* combination (GA).

A total of 315 healthy one-day-old male yellow-feather broiler chickens (*Lingnan yellow* chicken) were randomly assigned to 3 dietary treatment groups, with 7 replicates per group and 15 chickens per replicate. Each replicate was considered an experimental unit. The control group (CON) was fed a basal diet, whereas the low-GA (LGA) and high-GA (HGA) groups received the same basal diet supplemented with 0.1 and 0.3% GA, respectively. The experiment was divided into three phases: starter (days 1–28), grower (days 29–56), and finisher (days 57–84). The basal diet was formulated following the “Nutrient requirements of yellow chickens” (NY/T 3645–2020, China), as shown in Table 1.

**Table 1:** Composition and nutrient content of the basal diets (as-fed basis, %).

Items	Starter 1–28 days of age	Grower 29–56 days of age	Finisher 57–84 days of age
Ingredients			
Corn	51.7	50.7	58.7
Soybean oil	2.5	4.6	5.2
Soybean meal	28	26	23
Cottonseed meal	8	7.6	7
Rapeseed meal	0	8	3.36
Corn gluten meal	6.1	0	0
CaHPO_4_	1.5	0.82	0.6
NaCl	0.35	0.34	0.3
*L*-Lysine hydrochloride	0.2	0.36	0.2
*DL*-Methionine	0.1	0.1	0.1
Cysteine	0.08	0.08	0.03
Premix^a^	1.47	1.4	1.51
Total	100	100	100
Nutrient levels^b^			
ME, MJ/kg	12.39	12.61	12.83
Crude protein	21.10	17.50	16.09
Calcium	0.94	0.77	0.72
Total phosphorus	0.68	0.59	0.52
Available phosphorus	0.41	0.30	0.27
Lysine	1.10	0.98	0.85
Methionine	0.47	0.44	0.40
Methionine + Cystine	0.79	0.73	0.63

a The premix provided the following per kg of diets: 1–28 days: VA 12,000 IU; VD_3_ 3,500 IU; VE 60 IU; VK_3_ 4 mg; VB_1_ 10 mg; VB_2_ 10 mg; VB_6_ 6 mg; VB_12_ 8 μg; *D*-pantothenic acid 40 mg; nicotinic acid 75 mg; folic acid 10 mg; biotin 0.8 mg; choline 700 mg; Zn 90 mg; Fe 110 mg; Cu 20 mg; Mn 100 mg; I 0.5 mg; Se 0.3 mg. 29–56 days: VA 11,000 IU; VD_3_ 3,300 IU; VE 55 IU; VK_3_ 3.5 mg; VB_1_ 6 mg; VB_2_ 10 mg; VB_6_ 5 mg; VB_12_ 6 μg; *D*-pantothenic acid 30 mg; nicotinic acid 70 mg; folic acid 9 mg; biotin 0.7 mg; choline 600 mg; Zn 80 mg; Fe 100 mg; Cu 17 mg; Mn 90 mg; I 0.5 mg; Se 0.3 mg. 57–84 days: VA 10,000 IU; VD_3_ 3,000 IU; VE 50 IU; VK_3_ 3.0 mg; VB_1_ 2 mg; VB_2_ 14 mg; VB_6_ 5 mg; VB_12_ 4 μg; *D*-pantothenic acid 20 mg; nicotinic acid 60 mg; folic acid 7 mg; biotin 0.6 mg; choline 600 mg; Zn 70 mg; Fe 100 mg; Cu 15 mg; Mn 85 mg; I 0.5 mg; Se 0.3 mg.

b Metabolizable energy (ME) Available phosphorus (AP), and amino acid contents were calculated value, and the other nutrient levels were measured values.

The experiment was carried out at *ShunHe* Broiler Breeding Farm (Lanzhou, China). Broilers were raised in a flat net-rearing system (70 cm above the concrete floor), with each replication housed in a separate pen (200 × 100 cm) constructed with a stainless-steel frame and a flat wire mesh cover. The broilers were kept in an enclosed room with a ventilation regime and wet curtain cooling system. The experiment was conducted under the conditions of room temperature, lighting regime and immunization schedule described by Li et al. (24). The experiment lasted for 84 days, with ad libitum access to feed and water.

### 2.2 Growth and carcass performance

All birds were weighed after fasting overnight every 2 weeks, and the feed intake (FI) per replicate was recorded daily. The average daily gain (ADG), average daily feed intake (ADFI) and feed-to-gain ratio (F/G) were calculated. Mortality was recorded as it occurred, and performance parameters were adjusted accordingly.

On day 84, two broilers were randomly selected from each replicate using a random number table and slaughtered after fasting overnight. After bleeding and defeathering, individual carcass weights were recorded. Systematic evisceration was performed, and the organ and tissue weights were measured according to standardized protocols. The left pectoral muscle was sampled to assess meat quality. The dressing, semi-eviscerated, eviscerated, abdominal fat, pectoral muscle, and leg muscle rates were calculated following the “Technical Specification for Performance Testing of Meat-Type Chicken” (NY/T 828-2025, China).

### 2.3 Sample collection

On days 28, 56, and 84, following an overnight fast, two broilers were randomly selected from each replicate via a random number table, with a total of 14 broilers per group. Blood samples were collected from the wing vein of each individual, and serum was separated by centrifugation at 3,000 rpm for 15 min at 4 °C. The thymus, spleen, and bursa of Fabricius were then excised, weighed, and their relative organ weights (g/kg) calculated by dividing each organ’s weight by the live body weight. Sections of 1.5 cm from the middle of the duodenum, jejunum, and ileum were excised and fixed in 4% paraformaldehyde after removing the contents. The mucosa of remaining jejunum and the cecal content were gently scraped into a sterile tube and stored at −80 °C until analysis.

### 2.4 Meat quality

The left pectoral muscle was sampled to assess meat quality following the “Determination of Livestock and Poultry Meat Quality” (NY/T 1333–2007, China) standard. pH was measured using a pH meter (Mettler Toledo, Zurich, Switzerland), while color lightness (L*), redness (a*), and yellowness (b*) were measured using a colorimeter (Minolta, Tokyo, Japan) at 45 min and 24 h post-slaughter. Cooking loss was determined by cutting meat samples into 3 × 3 cm pieces, steaming them for 30 min, and re-weighing after cooling to room temperature. The cooking loss was calculated as the percentage of weight lost during cooking. Shear force was measured using a skinless meat sample (1 cm thick and 2.5 cm in diameter) cooked to an internal temperature of 70 °C. The force required to shear the sample was recorded with a tenderness tester (C-LM3B, Tenovo, Beijing, China).

### 2.5 Immune factors

Immunoglobulin A (IgA) (SEKCN-0018), IgG (SEKCN-0126), IgM (SEKCN-0128), interleukin (IL)-2 (SEKCN-0007), IL-10 (SEKCN-0097) and tumor necrosis factor (TNF)-α (SEKCN-0006) concentrations in serum were measured using commercial enzyme linked immunosorbent assay (ELISA) kits (Solarbio Science & Technology Co., Beijing, China) following the manufacturer’s instructions. Secretory immunoglobulin A (sIgA) content in jejunal mucosa was measured using the ELISA kit (AD72399; Wuhan Adanti Biological Technology Co., Ltd., China).

### 2.6 Intestinal histomorphology

The intestinal samples were fixed in 4% paraformaldehyde solution for 72 h, sectioned according to standard protocols, and then stained with hematoxylin and eosin (HE). Images were examined by optical microscope (Eclipse Ci-L, Nikon, Japan). Villus height (VH) and crypt depth (CD) were measured at three separate locations using Image Pro Plus 6.0 (Media Cybernetics, Bethesda, MD, United States), and the VH/CD ratio was then calculated.

### 2.7 Real-time PCR analysis

The total RNA was isolated from jejunal mucosa samples using Trizol reagent (TaKaRa, Japan). The integrity of RNA was assessed by electrophoresis on 0.8% agarose gels, while the purity and concentration were measured using a NanoDrop 1000 ultra micro spectrophotometer (Thermo Fisher Scientific, United States). cDNA was synthesized using the PrimeScript™ RT Master Mix reverse transcription kit (Takara Bio, Japan). qRT-PCR was performed using TB Green Premix Ex Taq TM (Tli RNaseH Plus) kit (TaKaRa Bio, China) (13). Relative quantification was performed using the 2^−△△Ct^ method, normalized to *β-actin* as reference. Primer sequences are provided in Table 2.

**Table 2:** Primer sequences used to measure gene expression.

Gene	Accession number	Primer sequences (5′ → 3′)	Product size, bp
*β-actin*	NM_205518.2	F: TCCACCGCAAATGCTTCTAA R: AAGCCATGCCAATCTCGTCT	104
Occludin (*OCLN*)	NM-205128.1	F: ACGGCAGCACCTACCTCAA R: GGGCGAAGAAGCAGATGAG	123
Zonula occluden-1 (*ZO-1*)	XM-015278980.4	F: CTTCAGGTGTTTCTCTTCCTCCTC R: CTGTGGTTTCATGGCTGGATC	131
Claudin-1 (*CLDN1*)	NM_001013611.2	F: TACTCCTGGGTCTGGTTGGT R: GTGCTGACAGACCTGCAATG	138

### 2.8 16S rRNA sequencing of cecal microbiota

Bacterial genomic DNA was extracted individually from the cecal contents of six broilers per group using the TGuide S96 kit, followed by purity and quality assessment through 0.8% agarose gel electrophoresis. The complete 16S rRNA gene was PCR-amplified using the primers (27F, AGRGTTTGATYNTGGCTCAG) and (1492R, TASGGHTACCTTGTTASGACTT). Following purification, quantification, and homogenization, the PCR products were utilized to construct a sequencing library on the PacBio platform (Biomarker Technologies Co., China). SMRT-Link v8.0 software was used to obtain Circular Consensus Sequencing (CCS) sequences. CCS sequences were identified using Lima v1.7.0, and chimeras were removed using UCHIME v4.2. The generated datasets were analyzed using USEARCH v10.0, where high-quality sequences were categorized into operational taxonomic units (OTUs) based on 97% similarity. BMK Cloud^1^ was used for microbial diversity and composition analyses (25).

### 2.9 Untargeted metabolomics of cecum content

A 50 mg sample of cecal content from each of six broilers per group was homogenized with an extraction solution (methanol: acetonitrile: water = 2:2:1, v/v/v) by vortexing for 30 s. The mixture was then added to porcelain beads, ground at 45 Hz for 10 min, and ultrasonicated for 10 min on an ice bath. After storage at −20 °C for 1 h, the samples were centrifuged at 12,000 × g (4 °C) for 15 min. The supernatant (120 μL per sample) was collected in 2 mL injection vials for analysis, and 10 μL aliquots were pooled to create a quality control (QC) sample (26). Metabolite profiling was conducted using an Acquity I-Class PLUS UHPLC system (Waters Corp., Milford, MA, United States) coupled with a Xevo G2-XS QTof high-resolution mass spectrometer equipped with an Acquity UPLC HSS T3 column (1.8 μm, 2.1 × 100 mm; Waters Corp., Taunton, MA, United States). The obtained compounds were identified by searching the MS/MS spectra against both the Human Metabolome Database (HMDB^2^) and METLIN database.^3^ The processed data were subsequently uploaded to the BMK Cloud^4^ for comprehensive analysis. Differential metabolites (DMs) were chosen based on Variable Importance in Projection (VIP) scores from orthogonal partial least squares-discriminant analysis (OPLS-DA) modeling (> 1.0) and statistical significance (*p* < 0.05) as determined by Student’s *t*-test. KEGG pathway enrichment analysis^5^ was performed to identify metabolic pathways that were significantly enriched with DMs.

### 2.10 Statistical analysis

Data were analyzed using SPSS software 26.0 (IBM Corp., NY, United States). Normality of data distribution was assessed via the Shapiro–Wilk test, and homogeneity of variances was verified using Levene’s test. For growth performance, carcass traits, meat quality, and immune organ index, one-way analysis of variance (ANOVA) was performed with each pen as the experimental unit, followed by Duncan’s post-hoc test for multiple comparisons among CON, LGA, and HGA. Given the significantly improved growth performance in the HGA group observed in the primary analysis, a pre-specified targeted assessment of serum immune indices and intestinal morphology was conducted only in the CON and HGA groups, with each broiler serving as an independent experimental unit. Differences between these two groups were evaluated using the unpaired Student’s *t*-test. Spearman’s correlation analysis was used to determine the relationship between the cecal microbiota and metabolites. Data are presented as the mean and standard error of the mean (SEM). Statistical significance was set at *p*-value < 0.05. GraphPad Prism 8.0 software (GraphPad, Inc., San Diego, CA, United States) was used for data visualization.

## 3 Results

### 3.1 HGA enhances growth performance in the grower-finisher stage

The broilers remained healthy, and no disease occurred throughout the experiment. No significant differences (*p* > 0.05) were observed in BW on days 1 and 28, as well as in ADG, ADFI, and F/G from days 1 to 28 among the groups (Table 3). Compared to the CON group, LGA significantly increased ADG and decreased (*p* < 0.05) F/G from days 29 to 56, but no difference was observed in any of the tested measures from days 57 to 84 or days 1 to 84. However, HGA significantly increased (*p* < 0.05) BW on days 56 and 84, and ADG from days 29 to 56, days 57 to 84, and days 1 to 84, while significantly decreasing F/G (*p* < 0.05). Moreover, compared to the LGA group, the HGA group significantly increased ADG from days 29 to 56 and days 57 to 84, while decreasing F/G from days 57 to 84 and days 1 to 84. These results indicate that supplementation with 0.3% GA (HGA) enhances growth performance in broilers, particularly during the grower-finisher stage.

**Table 3:** Effects of GA supplementation on the growth performance in broiler chickens.

Items	CON	LGA	HGA	SEM	*P-*value
Day 1–28					
BW (1 d), g	34.87	34.91	34.90	0.155	0.981
BW (28 d), g	717.77	703.82	718.14	5.517	0.561
ADFI, g/d	44.53	43.68	44.27	0.527	0.516
ADG, g/d	24.39	23.89	24.44	0.163	0.059
F/G (feed/gain)	1.83	1.83	1.81	0.031	0.911
Day 29–56					
BW (56 d), g	2077.89^c^	2118.28^b^	2136.05^a^	4.308	0.001
ADFI, g/d	133.61	132.37	133.01	0.984	0.677
ADG, g/d	48.58^b^	50.52^a^	50.64^a^	0.363	0.002
F/G	2.75^a^	2.62^b^	2.63^b^	0.024	0.002
Day 57–84					
BW (84 d), g	3186.59^c^	3242.80^b^	3320.20^a^	6.948	0.001
ADFI, g/d	164.37	171.29	167.05	2.068	0.090
ADG, g/d	39.60^b^	40.16^b^	42.29^a^	0.271	0.001
F/G	4.15^a^	4.27^a^	3.95^b^	0.044	0.001
Day 1–84					
ADFI, g/d	114.17	115.78	114.78	36.772	1.001
ADG, g/d	37.52^b^	38.19^ab^	39.12^a^	0.301	0.026
F/G	3.04^a^	3.03^a^	2.93^b^	0.024	0.042

CON, control diet; LGA, diet supplemented with 0.1% *Glycyrrhiza-Atractylodes* combination; HGA, diet supplemented with 0.3% *Glycyrrhiza-Atractylodes* combination; BW, body weight; ADFI, average daily feed intake; ADG, average daily gain; F/G, feed/gain.

^a,b,c^Values with the same or no letter superscripts in the same row indicate no significant difference (*p* > 0.05), while those with different letter superscripts indicate significant difference (*p* < 0.05) (*n* = 7).

### 3.2 HGA improves carcass performance

Compared to the CON group, LGA significantly increased (*p* < 0.05) leg muscle rate, while no significant difference (*p* > 0.05) was observed in other carcass performance indicators (Table 4). The HGA group had higher dressing, semi-eviscerated, eviscerated, and leg muscle rates, as well as a lower abdominal fat rate (*p* < 0.05). These results suggest that HGA improves carcass performance of broilers.

**Table 4:** Effects of GA supplementation on carcass performance in broilers on day 84.

Items, %	CON	LGA	HGA	SEM	*P-*value
Dressing rate	90.58^b^	91.90^ab^	93.44^a^	0.681	0.028
Semi-eviscerated rate	83.51^b^	84.34^b^	86.95^a^	0.687	0.006
Eviscerated rate	65.53^b^	66.11^b^	68.13^a^	0.578	0.013
Breast muscle rate	14.29	15.59	14.54	0.590	0.282
Leg muscle rate	23.57^b^	25.21^a^	25.46^a^	0.563	0.001
Abdominal fat rate	3.05^a^	3.20^a^	2.59^b^	0.173	0.047

CON, control diet; LGA, diet supplemented with 0.1% *Glycyrrhiza-Atractylodes* combination; HGA, diet supplemented with 0.3% *Glycyrrhiza-Atractylodes* combination.

^a,b^Values with the same or no letter superscripts in the same row indicate no significant difference (*p* > 0.05), while those with different letter superscripts indicate significant difference (*p* < 0.05) (*n* = 14).

### 3.3 HGA improves meat quality

Compared to the CON group, both LGA and HGA did not affect (*p* > 0.05) pH and cooking loss of the breast muscle. However, LGA significantly decreased (*p* < 0.01) L*_45min_ and b*_45min_, while HGA decreased (*p* < 0.01) L*_45min_, b*_45min_, L*_24h_, and shear force, and increased a*_45min_ and a*_24h_ of the breast muscle (Table 5).

**Table 5:** Effects of GA supplementation on meat quality of breast muscle in broilers on day 84.

Items	CON	LGA	HGA	SEM	*P-*value
pH_45min_	5.66	5.53	5.55	0.075	0.434
pH_24h_	5.54	5.40	5.48	0.062	0.334
L*_45min_	40.47^a^	38.61^b^	36.95^b^	0.620	0.003
b*_45min_	1.24^a^	1.01^b^	0.96^b^	0.054	0.007
a*_45min_	3.65^b^	3.71^b^	4.12^a^	0.078	0.001
L*_24h_	39.62^a^	39.71^a^	37.64^b^	0.433	0.009
b*_24h_	1.26	1.14	1.09	0.046	0.052
a*_24h_	3.79^b^	3.89^ab^	4.04^a^	0.062	0.037
Cooking loss, %	28.44	25.84	25.09	1.220	0.155
Shear force, *N*	47.08^a^	46.26^ab^	44.78^b^	0.520	0.018

CON, control diet; LGA, diet supplemented with 0.1% Glycyrrhiza-Atractylodes combination; HGA, diet supplemented with 0.3% Glycyrrhiza-Atractylodes combination; L*, lightness; b*, yellowness; a*, redness.

^a,b^Values with the same or no letter superscripts in the same row indicate no significant difference (*P* > 0.05), while those with different letter superscripts indicate significant difference (*P* < 0.05) (*n* = 14).

### 3.4 HGA promotes the development of immune organs

Compared to the CON group, both LGA and HGA did not affect (*p* > 0.05) the immune organ index of broilers on day 28 (Table 6). However, LGA significantly increased (*p* < 0.05) the bursa of Fabricius index on days 56 and 84, while significantly HGA increased the thymus and bursa of Fabricius indices on days 56 and 84 (*p* < 0.05).

**Table 6:** Effects of GA supplementation on organ index in broiler chickens (g/kg).

Items	CON	LGA	HGA	SEM	*P-*value
Day 28					
Thymus	3.10	3.13	3.24	0.061	0.232
Spleen	1.55	1.57	1.49	0.076	0.781
Bursa of Fabricius	3.11	3.18	3.21	0.083	0.631
Day 56					
Thymus	2.16^b^	2.27^ab^	2.31^a^	0.038	0.031
Spleen	1.24	1.35	1.30	0.034	0.110
Bursa of Fabricius	0.80^b^	1.09^a^	0.97^a^	0.053	0.042
Day 84					
Thymus	1.04	1.12	1.15	0.041	0.178
Spleen	1.11	1.16	1.24	0.082	0.524
Bursa of Fabricius	0.63^b^	0.74^a^	0.71^b^	0.052	0.025

CON, control diet; LGA, diet supplemented with 0.1% Glycyrrhiza-Atractylodes combination; HGA, diet supplemented with 0.3% Glycyrrhiza-Atractylodes combination.

^a,b^Values with the same or no letter superscripts in the same row indicate no significant difference (*P* > 0.05), while those with different letter superscripts indicate significant difference (*P* < 0.05) (*n* = 14).

### 3.5 HGA regulates serum immune and inflammatory factors

**Figure 1:**
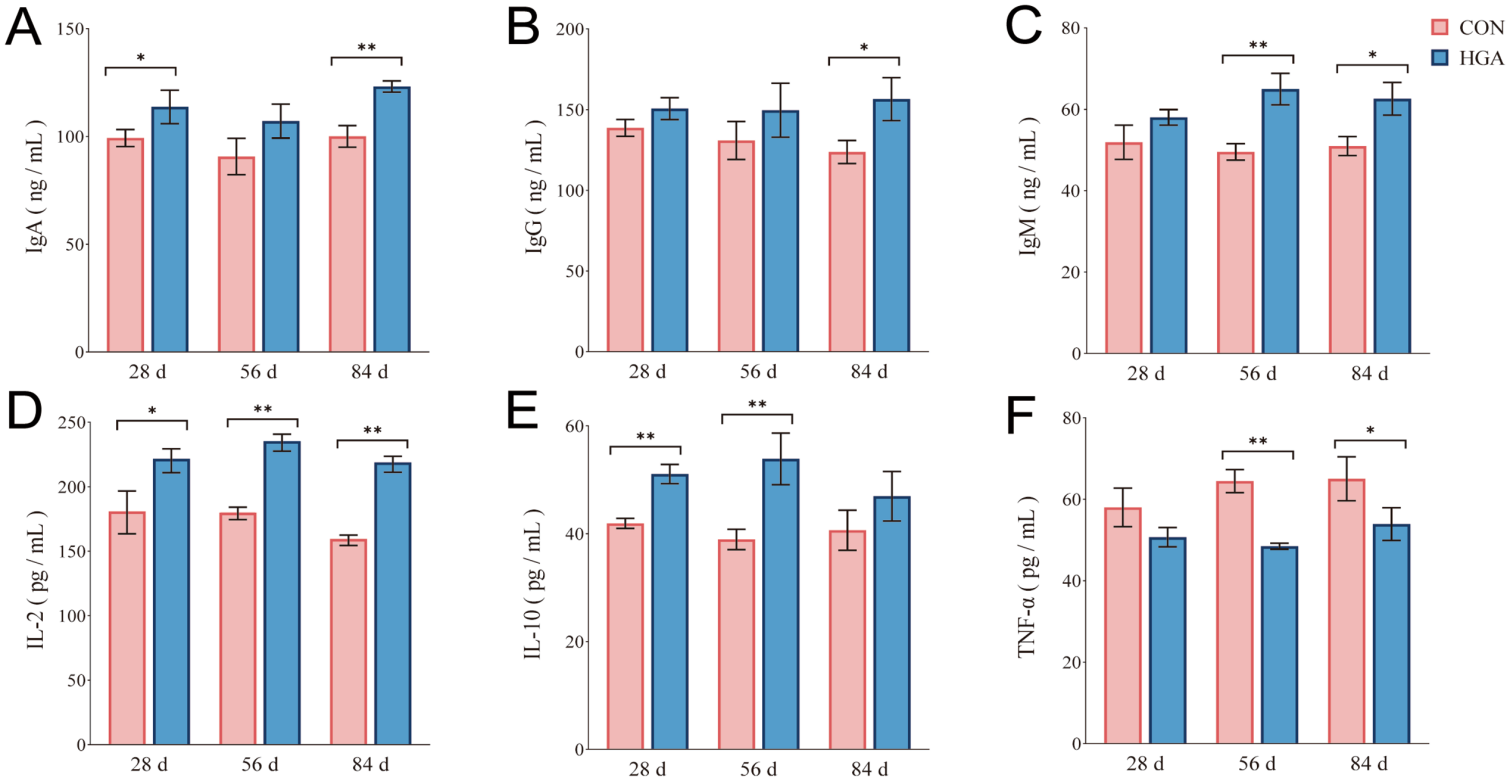
Effects of HGA supplementation on serum immune and inflammatory factors in broilers. **(A)** Immunoglobulin A (IgA). **(B)** IgG. **(C)** IgM. **(D)** Interleukin (IL)-2. **(E)** IL-10. **(F)** Tumor necrosis factor (TNF)-α. *p < 0.05, **p < 0.01, *n* = 14.

Based on the findings on growth performance and immune organs, the HGA group was selected to evaluate variations in serum levels of immune and inflammatory factors (Figure 1). Compared to the CON group, HGA significantly increased (*p* < 0.05) serum IgA, IL-2, and IL-10 levels on day 28; increased IgM, IL-2, and IL-10 levels while reducing TNF-α levels on day 56; and increased IgA, IgG, IgM, and IL-2 levels while reducing TNF-α levels on day 84. This indicates that HGA supplementation can enhance immunity in broilers.

### 3.6 HGA improves intestinal morphology and barrier function

**Figure 2:**
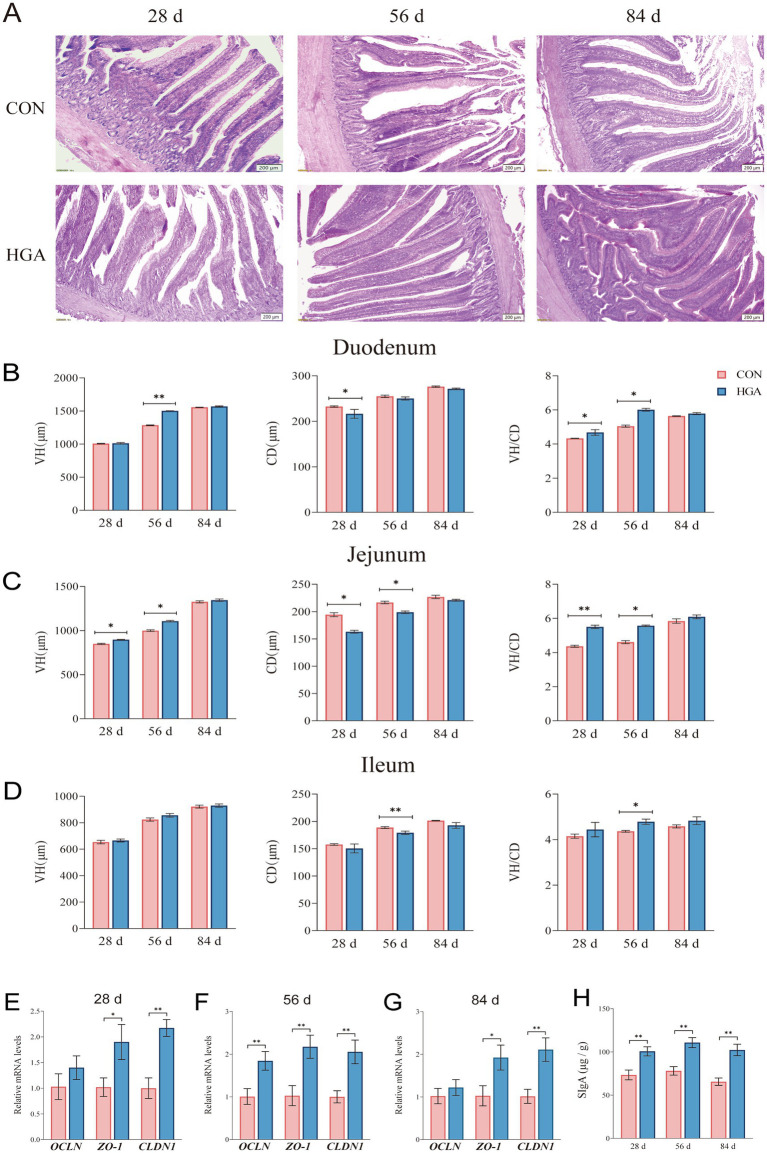
Effects of HGA on intestinal morphology and barrier function. **(A)** Representative HE images for jejunum tissues (scale bars represent 200 μm). **(B)** Duodenum morphology. **(C)** Jejunum morphology. **(D)** Ileum morphology. VH, villi height. CD, crypt depth. **(E–G)** The mRNA expression of intestinal barrier-related genes in the jejunum mucosa. *OCLN*, occludin. *ZO-1*, zonula occluden-1. *CLDN1*, claudin-1. **(H)** The secretory immunoglobulins A (sIgA) levels in the jejunum mucosa. *p < 0.05, ** *p* < 0.01, *n* = 14.

The intestinal morphology of broilers was presented in Figures 2A–D. At day 28, jejunum morphology was normal with well-organized villi and intact crypts, whereas thin intestinal walls and underdeveloped villi were observed in the two groups, suggesting immaturity in early intestinal development. At days 56 and 84, the normal architecture was maintained, and no acute or chronic damage was observed. Compared to the CON group, HGA significantly increased (*p* < 0.05) jejunal VH, VH/CD, and duodenal VH/CD on day 28, while reducing duodenal and jejunal CD. HGA significantly increased (*p* < 0.05) duodenal and jejunal VH and VH/CD, and ileal VH and VH/CD on day 56, while reducing jejunal and ileal CD. However, no significant differences were observed on day 84 (*p* > 0.05). These results demonstrated that HGA promoted intestinal morphological development, specifically during the early growth stages of broilers.

Compared to the CON group, HGA significantly increased (*p* < 0.05) the expression of *ZO-1* and *CLDN1* in the jejunum mucosa of broilers at days 28, 56 and 84, and increased *OCLN* expression at day 56. Additionally, it significantly increased (*p* < 0.01) the sIgA content in the jejunum mucosa (Figures 2E–H). This indicated that HGA improved intestinal barrier function.

### 3.7 Cecum microbiota

#### 3.7.1 HGA alters the diversity and structure of the cecal microbiota

**Figure 3:**
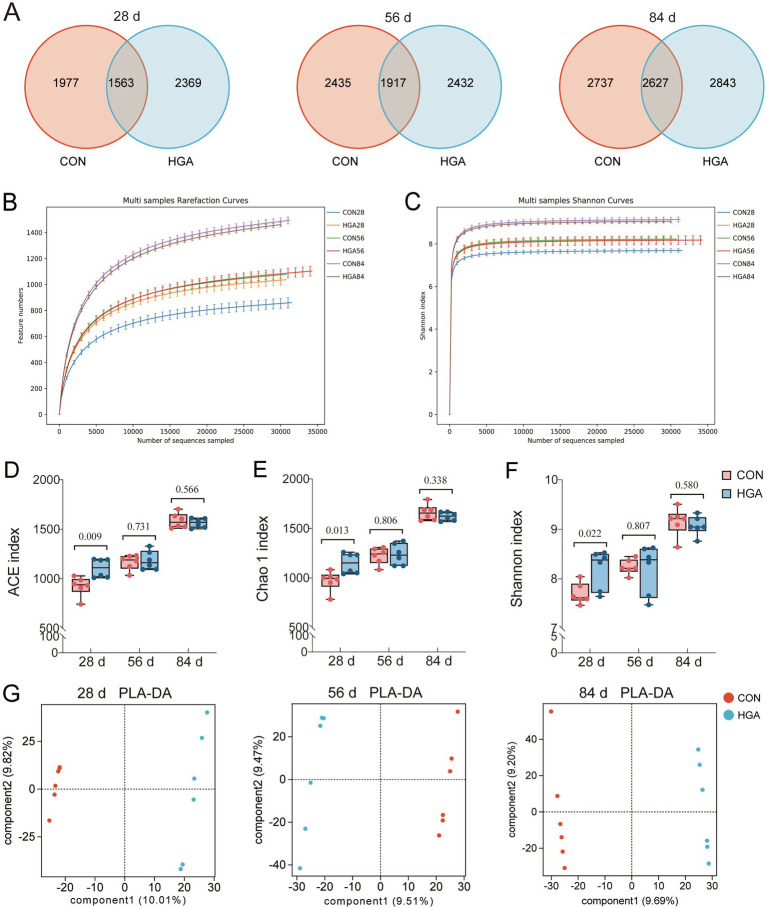
Effects of HGA on cecal microbiota diversity in broiler. **(A)** Venn diagram. **(B)** Rarefaction curve. **(C)** Shannon-Wiener curve. **(D)** ACE index. **(E)** Chao 1 index. **(F)** Shannon index. **(G)** PLS-DA based on OTUs, *n* = 6.

To investigate the impact of HGA on broiler intestinal microbiota, 16S rRNA sequencing was performed on cecal chyme samples. The rarefaction and Shannon-Wiener curves confirmed the sufficient sample size and sequencing depth for all samples, as evidenced by the saturation plateau (Figures 3B,C). The Venn diagram showed unique operational taxonomic unit (OTU) counts of 1,977 (CON) vs. 2,369 (HGA) at day 28, 2,435 vs. 2,432 at day 56, and 2,737 vs. 2,843 at day 84 (Figure 3A).

Compared to the CON group, HGA significantly increased (*p* < 0.05) the ACE, Chao 1, and Shannon indices on day 28, while no significant differences (*p* > 0.05) were noted on days 56 and 84 (Figures 3D–F). This indicated that HGA increased the richness and diversity of the cecal microbiota in broilers during the early growth stage. Beta diversity was visualized through partial least squares discriminant analysis (PLS-DA) based on sample distances. The PLS-DA results revealed distinct clustering patterns between groups, with samples from each group forming tight clusters at all three time points (Figure 3G), suggesting that HGA significantly altered the cecal microbiota composition of broilers.

#### 3.7.2 HGA induces age-dependent alterations in cecal microbiota composition at phylum and genus levels

**Figure 4:**
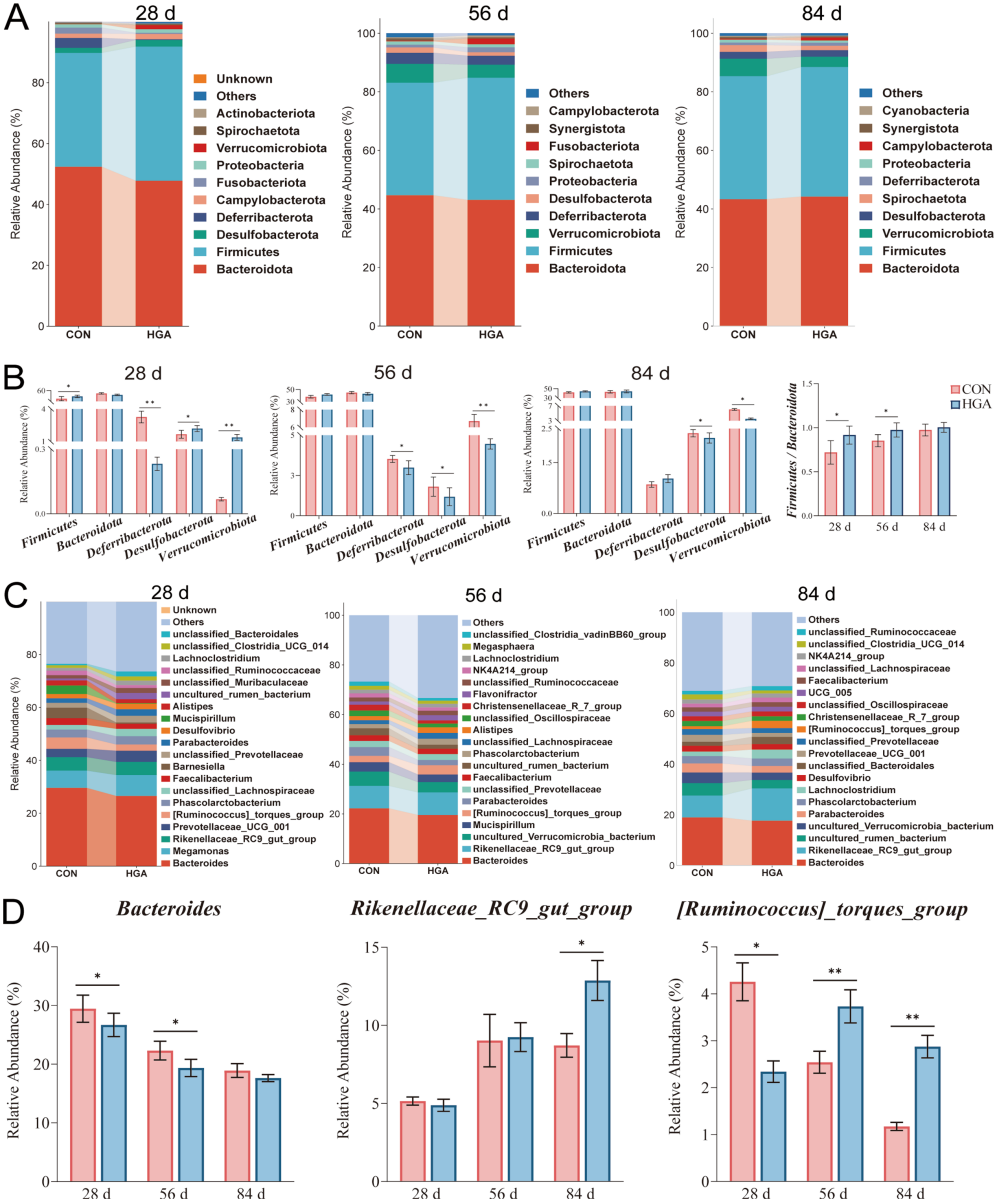
The relative abundance of taxa in the cecal microbiota at the phylum and genus levels. **(A)** Relative abundance taxa at the phylum level. The top 10 phyla by relative abundance were shown, while the remaining phyla were categorized as “Others.” **(B)** The relative abundance of dominant bacterial phyla. **(C)** Relative abundance taxa at the genus level. The top 20 genera by relative abundance were shown, while the remaining genera were categorized as “Others.” **(D)** The relative abundance of dominant bacterial genera. *p < 0.05, **p < 0.01, *n* = 6.

A total of 12 microbial phyla were identified in the cecum microbiota of broilers in the two groups (Figure 4A). The dominant bacterial phyla with relative abundance greater than 3% across all three growth stages of broilers included *Bacteroidota*, *Firmicutes*, *Desulfobacterota*, *Deferribacterota*, and *Verrucomicrobiota*. Compared to the CON group, HGA significantly increased (*p* < 0.05) the relative abundances of *Firmicutes*, *Desulfobacterota*, and *Verrucomicrobiota* and reduced that of *Deferribacterota* on day 28 (Figure 4B). On day 56, it increased the abundance of *Firmicutes*, whereas it reduced *Verrucomicrobiota*, *Deferribacterota*, and *Desulfobacterota*. By day 84, *Verrucomicrobiota* and *Desulfobacterota* decreased in the HGA group (*p* < 0.05). Additionally, HGA significantly increased the *Firmicutes*/*Bacteroidota* (F/B) ratio on days 28 and 56.

The top 20 genera by relative abundance in the cecal microbiota were presented in Figure 4C. The dominant bacterial genera with relatively high abundance across all three growth stages of broilers included *Rikenellaceae_RC9_gut_group*, *[Ruminococcus]_torques_group*, and *Bacteroides*. Compared with the CON group, HGA significantly reduced the relative abundance of *Bacteroides* and *[Ruminococcus]_torques_group* on day 28 (*p* < 0.05); increased *[Ruminococcus]_torques_group* (*p* < 0.05), while reducing *Bacteroides* on day 56; and increased *Rikenellaceae_RC9_gut_group* and *[Ruminococcus]_torques_group* on day 84 (*p* < 0.05) (Figure 4D).

**Figure 5:**
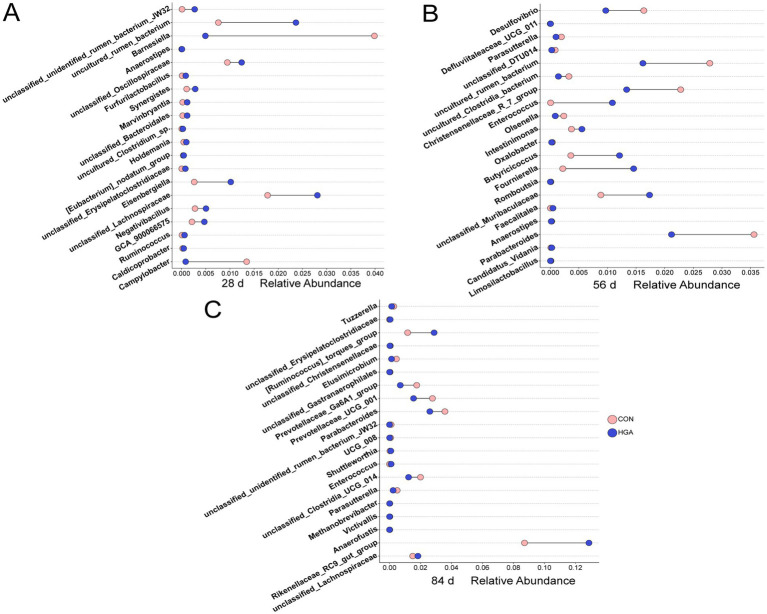
Dumbbell chart of random forest classification method analysis. **(A)** Day 28; **(B)** Day 56; **(C)** Day 84. Bacterial flora was classified and colored at the genus level, and the differential bacterial genera were sorted in descending order of importance to model accuracy. Pink dots indicate the CON group, and blue dots indicate the HGA group, *n* = 6.

A random forest classification method was applied to identify the biomarker bacteria driving structural differences between the two groups, using Mean Decrease Gini values and relative abundance as ranking criteria (Figure 5). On day 28, *uncultured_rumen_bacterium*, *unclassified_Oscillospiraceae*, *Eisenbergiella*, *unclassified_Lachnospiraceae*, *Negativibacillus*, and *Synergistes* were prominently increased bacterial genera, while *Barnesiella* and *Campylobacter* significantly decreased (*p* < 0.05) in the HGA group. On day 56, the relative abundance of *Enterococcus*, *Butyricicoccus*, *Fournierella*, and *unclassified_Muribaculaceae* was significantly increased, while *uncultured_rumen_bacterium*, *Desulfovibrio*, *Parasutterella* and *Christensenellaceae_R_7_group* were decreased (*p* < 0.05) in the HGA group. On day 84, *[Ruminococcus]_torques_group*, *Enterococcus*, and *Rikenellaceae_RC9_gut_group* significantly increased (*p* < 0.05), while *Prevotellaceae_UCG_001* and *Parasutterella* decreased in the HGA group. Overall, HGA altered the gut microbiota composition, suggesting a potential shift toward a healthier profile.

#### 3.7.3 HGA alters gut microbial interactions

**Figure 6:**
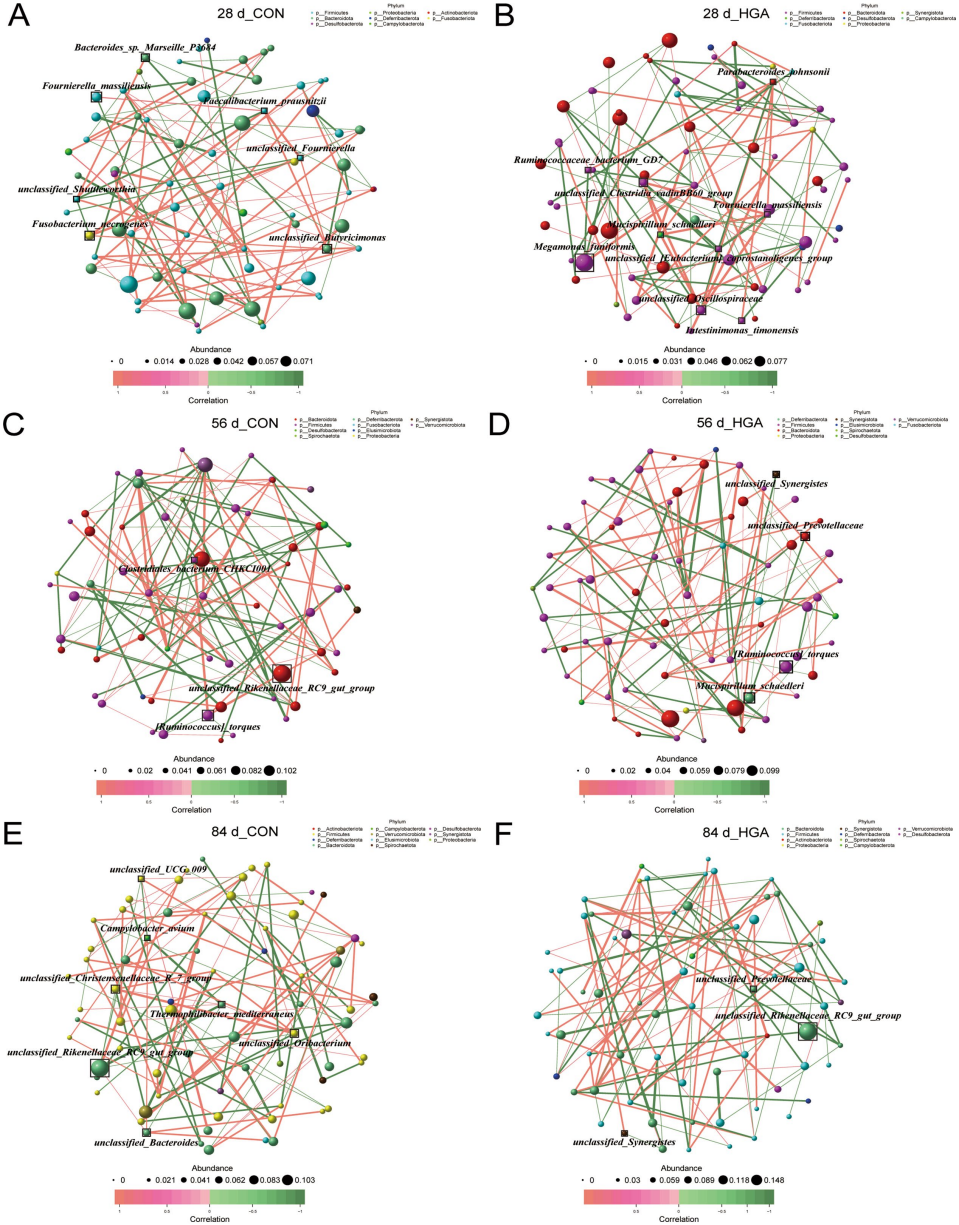
Microbial co-occurrence networks. **(A,B)** The CON and HGA groups at day 28. **(C,D)** The CON and HGA groups at day 56. **(E,F)** The CON and HGA groups at day 28. Spearman’s rank correlation analysis identified taxa interactions. Only significant correlations (*p* < 0.05, |*r*| > 0.5) were plotted. Node size reflects the number of significant inter-species connections, while node color indicates phylum. Edge color denotes correlation type: green (negative), pink (positive), *n* = 6.

Ecological network analysis was performed to assess the microbiota composition, network topology, and functional potential. The ecological networks were shown in Figure 6, and the average degree (AD) was used as an indicator of interaction intensity, whereas a larger Q value indicated greater stability of the microbial community function. On day 28, the CON group exhibited a microbial network with 95 nodes (AD = 4.8), divided into 8 modules (*Q* = 0.39). Among all the significant correlation edges, 74% were positively correlated and 25% were negatively correlated. *Faecalibacterium_brausnitzii*, as a core node with the highest degree of centralization and topological centrality, was significantly positively correlated with *Fusobacterium_necrogenes*, *unclassified_Butyricimonas*, and *Fournierella_massiliensis*, and negatively correlated with *Bacteroides_sp.*, *Marseille_P3684*, and *unclassified_Shuttleworthia*. In comparison, the HGA group exhibited 80 nodes (AD = 5.1) and 7 modules (*Q* = 0.41), with 71% positively correlated and 28% negatively correlated. The *Parabacteroides johnsonii*, as a core node, was positively correlated with *Ruminococcaceae_bacterium_GD7*, *Intestinimonas_timonensis*, *Mucispirillum_schaedleri*, and *Fournierella_massiliensis,* and negatively correlated with *Megamonas_funiformity* and *unclassified_[Eubacteria]_comprostantenes group* (Figures 6A,B).

On day 56, the CON group exhibited a microbial network with 80 nodes (AD = 5.2), and 6 modules (*Q* = 0.42), with 68% positively and 32% negatively correlated. *Unclassified_Rikenellaceae_RC9_gut_group*, as a core node, was positively correlated with *[Ruminococcus]_torques* and *Clostridiales_bacteria_CHKCI001*. The HGA group consisted of 68 nodes (AD = 5.3) and 6 modules (*Q* = 0.42), with 72% positively correlated and 28% negatively correlated. *Mucispirillum_stchaedleri* was the core node, positively correlated with *[Ruminococcus]_torques*, and negatively correlated with *unclassified_Prevotellaceae* and *unclassified_Synergistes* (Figures 6C,D).

On day 84, the CON group exhibited 74 nodes (AD = 3.08) and 7 modules (*Q* = 0.47), with 66 and 34% being positively and negatively correlated, respectively. The HGA group consisted of 80 nodes (AD = 5.42) and 12 modules (*Q* = 0.47), with 69% of the nodes positively correlated and 31% negatively correlated. The core nodes of both groups were *unclassified_Rikenellaceae_RC9_gut_group*. In the CON group, it was positively correlated with *Campylobacter_avium* and *unclassified_Bacteroides* and negatively correlated with *Thermophilibacter_mediterraneus*, *unclassified_UCG_009*, and *unclassified_Oribacterium.* In the HGA group, there was a negative correlation with *unclassified_Prevotellaceae* and *unclassified_Synergistes* (Figures 6E,F). Overall, HGA altered microbial association patterns reflecting potential shifts in community structure characterized by a denser, more interconnected network and a higher proportion of intense correlations at all three ages.

### 3.8 HGA exerts age-dependent regulation of cecum metabolic profiles

**Figure 7:**
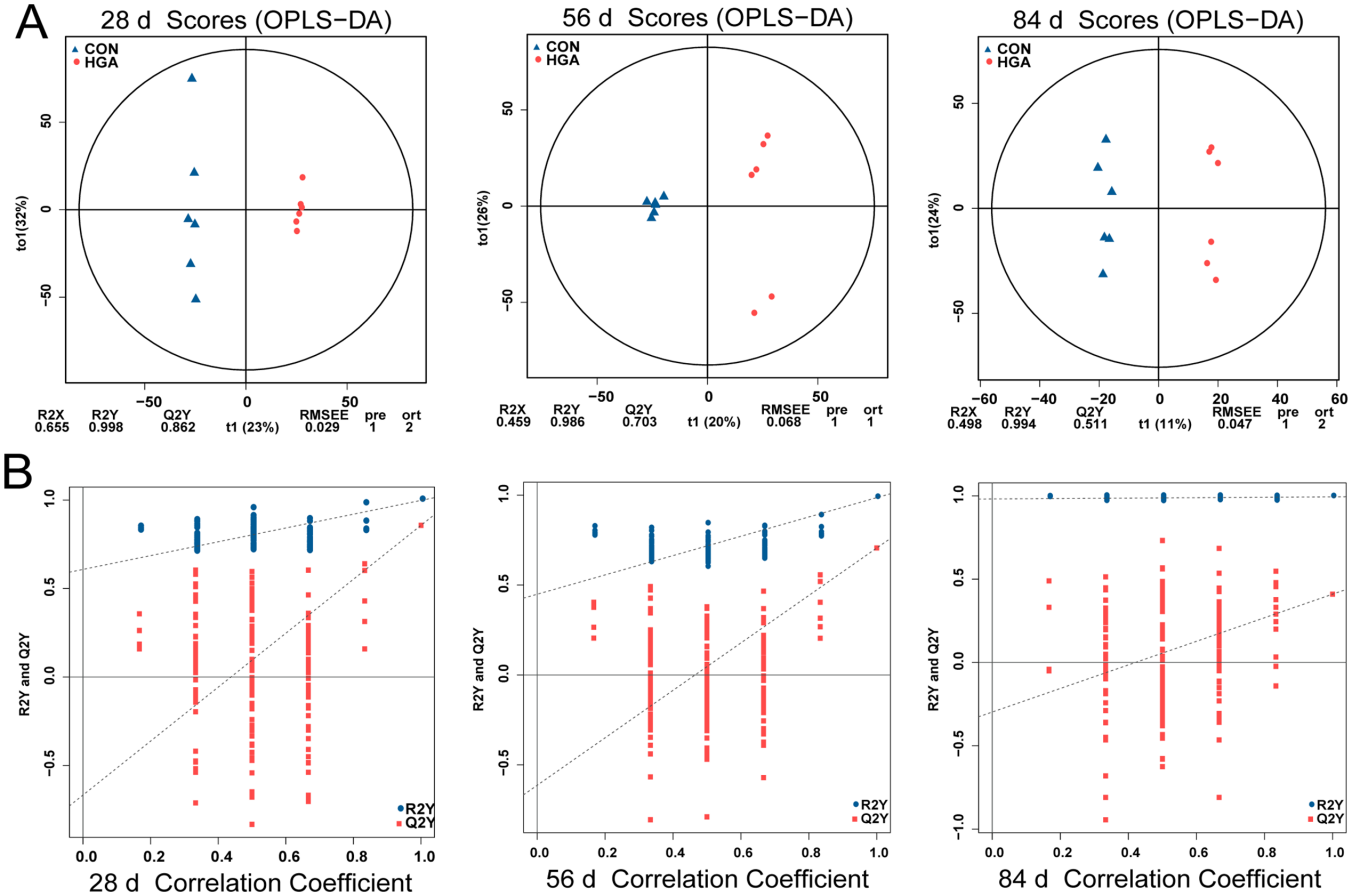
Orthogonal partial least squares-discriminant analysis (OPLS-DA) score plot. **(A)** OPLS-DA score plot. **(B)** Permutation test of the OPLS-DA model, *n* = 6.

To identify the potential metabolites and pathways induced by HGA, untargeted metabolomic analysis of cecal chyme samples was performed using UPLC-MS/MS. A total of 16,414 mass spectrum peaks were detected in samples across the two groups at three ages. Metabolites were putatively annotated (MSI level 2) by matching MS1 mass accuracy (<10 ppm) and MS/MS spectral similarity (>70%) against the HMDB database. 3,316 metabolites were annotated in the HMDB, comprising 1,518 positive ion metabolites and 1,798 negative ion metabolites. Raw *p*-values were adjusted using the Benjamini-Hochberg (BH) method to control false discovery rate (FDR). Orthogonal partial least squares-discriminant analysis (OPLS-DA) exhibited distinct group separation at 28 days (*R*^2^*Y* = 0.998), 56 days (*R*^2^*Y* = 0.986), and 84 days (*R*^2^*Y* = 0.994) (Figure 7). This indicated that HGA altered the metabolic profiles in the cecum of broilers, with progressive convergence with age.

**Figure 8:**
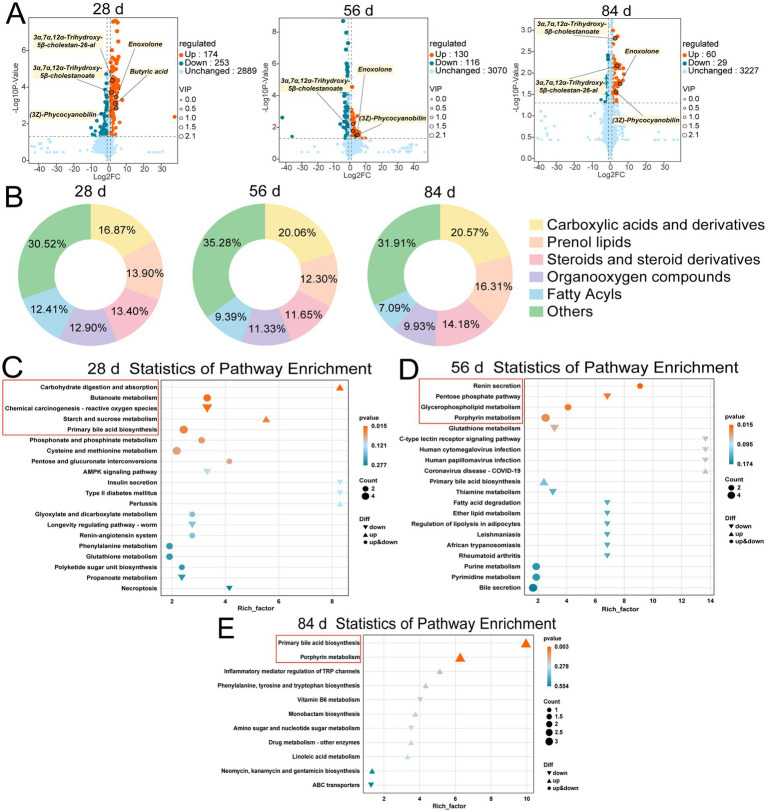
Effect of HGA on cecal metabolites in broiler. **(A)** Volcano plots. Variable importance in projection (VIP) > 1, *p* < 0.05, |log2FC| > 1. **(B)** Pie charts of top 5 metabolite categories with the highest number of differential metabolites (DMs). **(C–E)** KEGG pathway enrichment analysis of DMs, *n* = 6.

Differential metabolites (DMs) were defined as those with adjusted *p* < 0.05 and VIP > 1 from the OPLS-DA model. There were 427 DMs (174 upregulated and 253 downregulated), 246 DMs (130 upregulated and 116 downregulated), and 89 DMs (60 upregulated and 29 downregulated) identified in the HGA group on days 28, 56, and 84, respectively (Figure 8A). Notably, the levels of 3α, 7α, 12α-trihydroxy-5β-cholestanoate, enoxolone (glycyrrhetinic acid) and (3Z)-phycocyanobilin significantly increased over the three age intervals in the HGA group. The top five metabolite categories with the highest number of DMs were steroids and steroid derivatives, carboxylic acids and derivatives, fatty acyls, prenol lipids, and organooxygen compounds, accounting for 64.72–69.48% of the total DMs (Figure 8B).

KEGG enrichment analysis was conducted to further identify the metabolic pathways enriched by DMs (Figures 8C–E). Among these pathways, the porphyrin metabolism and primary bile acid biosynthesis were enriched by 3α, 7α, 12α-trihydroxy-5β-cholestanoate, 3α, 7α, 12α-trihydroxy-5β-cholestan-26-al, and (3Z)-phycocyanobilin at days 28, 56 and 84. These DMs were upregulated in the HGA group (*p* < 0.05). Moreover, on day 28, carbohydrate digestion and absorption and starch and sucrose metabolism were significantly upregulated by HGA, whereas chemical carcinogenesis-reactive oxygen species was downregulated. The carbohydrate digestion and absorption and butanoate metabolism pathways were enriched by butyric acid, which was upregulated in the HGA group (*p* < 0.05). Renin secretion, pentose phosphate pathway, and glycerophospholipid metabolism were significantly enriched on day 56. These results demonstrated that HGA exerted age-dependent regulation of gut microbial metabolism in broilers, with more pronounced effects during the early growth stage.

### 3.9 Correlation between cecal differential microbiota and DMs

**Figure 9:**
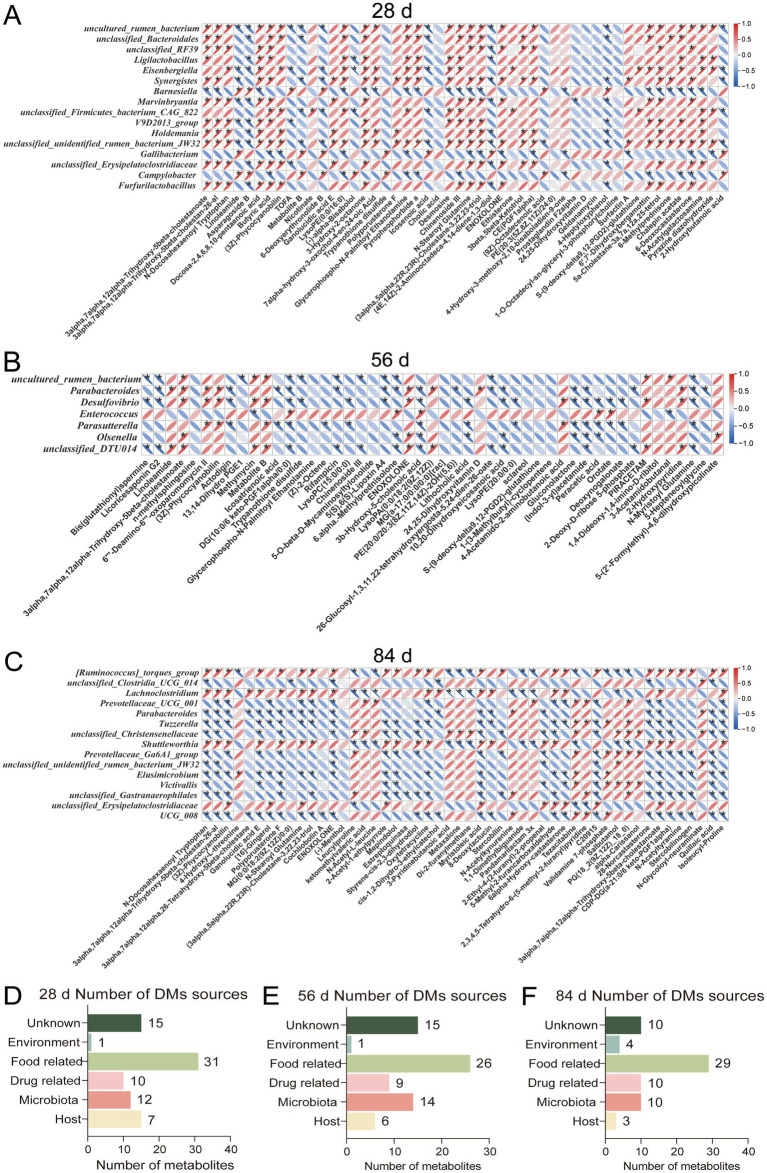
Correlation heatmap between differential metabolites (DMs) and differential microbiota, with source analysis of DMs. **(A–C)** Spearman correlation heatmap. Red indicates positive correlation, and blue indicates negative correlation. The intensity of the color indicates the strength of the correlation, and *p < 0.05. **(D–F)** Source of DMs, *n* = 6.

We integrated the cecal microbiome and metabolome using Spearman correlation analysis (|*r*| ≥ 0.6, *p* < 0.05) to further investigate the potential mechanism by which HGA affects broilers. As shown in Figures 9A–C, 50 DMs exhibited significant correlations with the cecal microbiota. MetOrigin analysis^6^ further revealed that these DMs originated from multiple sources, including host intestinal metabolism, microbiota, drugs, and feed (Figures 9D–F).

Enoxolone, the drug metabolite of glycyrrhizin, was significantly positively correlated (*p* < 0.05) with *Marvinbryantia*, *Synergistes*, *unclassified_unidentified_rumen_bacterium_JW32*, *Furfurifactobacillus*, and *unclassified_Erysipelatoclostridiaceae* on day 28, *Parabacteroides* and *Olsenella* on day 56, and *[Ruminococcus]_torques_group* and *Lachnoclostridium* on day 84, while negatively correlated with *Gallibacterium* on day 28, *Tuzzerella* and *Prevotellaceae_Ga6A1_group* on day 84. (3Z)-phycocyanobilin, the microbial metabolite, was positively correlated (*p* < 0.05) with *uncultured_rumen_bacterium*, *unclassified_Firmicutes_bacterium_CAG_822*, *unclassified_unidentified_rumen_bacterium_JW32*, *Holdemania*, and *unclassified_Erysipelatoclostridiaceae* on day 28, *Parabacteroides* on day 56, and *[Ruminococcus]_torques_group*, *Lachnoclostridium*, *Shuttleworthia*, and *unclassified_Erysipelatoclostridiaceae* on day 84, while negatively correlated with *Gallibacterium* on day 28, and *Tuzzerella* and *Prevotellaceae_Ga6A1_group* on day 84. Butyric acid, 3α, 7α, 12α-trihydroxy-5β-cholestanoate, and 3α, 7α, 12α-trihydroxy-5β-cholestan-26-al were host metabolites. Butyric acid was positively correlated (*p* < 0.05) with *Ligilactobacillus*, *Synergistes*, *Marvinbryantia*, and *Furfurilactobacillus* and negatively correlated with *Gallibacterium* and *Campylobacter* on day 28. On day 28, 3α, 7α, 12α-trihydroxy-5β-cholestanoate and 3α, 7α, 12α-trihydroxy-5β-cholestan-26-al were positively correlated (*p* < 0.05) with *Synergistes*, *Marvinbryantia*, *Holdemania*, and *Furfurilactobacillus*, while negatively correlated with *Barnesiella*. On day 84, they were positively correlated with *[Ruminococcus]_torques_group*, *Lachnoclostridium*, and *Shuttleworthia* and negatively correlated with *Parabacteroides*, *Tuzzerella*, and *Elusimicrobium*.

## 4 Discussion

The TCM comprises various herbs with distinct functions that achieve synergistic effects while mitigating potential adverse reactions. Consequently, the primary pharmacological effects of these formulations vary considerably depending on their specific compositional profiles. Accumulating evidence has demonstrated that various TCM formulations exert positive effects in poultry. In this study, we selected two representative Chinese herbs, GU and AM, to develop a novel TCM additive, designated GA. This combination was formulated to harness their complementary properties to enhance the health and growth of broilers.

### 4.1 Growth performance

Our study demonstrated that supplementation with low-dose GA (0.1%, LGA) showed a stage effect on broiler growth, significantly increasing ADG and decreasing F/G only in birds aged 29–56 days. However, high-dose GA (0.3%, HGA) enhanced growth performance, particularly during the grower-finisher stage, as evidenced by the increased ADG and reduced F/G from days 29 to 56, days 57 to 84, and days 1 to 84. Moreover, HGA increased net meat yield and improved carcass quality by increasing dressing rate, eviscerated rate, leg muscle rate, and decreasing abdominal fat rate, whereas LGA increased the relative weight of leg muscles.

The effects of GA supplementation on production performance are dependent on GU, AM, and their bioactive compounds. Previous research has shown that GU extract enhanced growth performance and antioxidant capacity and improved hematological parameters and lipid profiles in broilers (27, 28). The growth-promoting effects of GU were likely mediated through improved feed palatability and enhanced digestive enzyme secretion (29). GU polysaccharides activated the somatotropic axis, as evidenced by increased growth hormone (GH) and insulin-like growth factor 1 (IGF-1) secretion, thereby promoting broiler growth (23). Additionally, GU polysaccharides stimulated hypothalamic neuropeptide Y (NPY) and agouti-related peptide (AgRP) synthesis and release, leading to improved feed intake and growth in broilers (30). While research on the direct effects of AM in broilers remains limited, studies on TCM formulations containing AM have demonstrated significant improvements in ADG and BW in broilers (31, 32). Furthermore, compound herbal additives combining GU and AM have been shown to enhance slaughter performance in geese by increasing slaughter rate and half-eviscerated rate (33), providing preliminary evidence for the synergistic potential of these two herbs in improving poultry production traits.

Meat quality is a key factor influencing consumers’ purchasing willingness. Tenderness is assessed using various indicators, where shear force indicates tenderness, a higher L* value reflects paler meat, an elevated a* value signifies a more intense red coloration, and an increased b* value suggests undesirable yellowness. Our results assessing meat quality showed that HGA improved meat quality by reducing breast muscle L* and b* values and shear force while increasing the a* value. Similarly, GU polysaccharides increased fiber density in broiler breast muscles, thereby reducing dehydration rates (23). Qiao et al. (34) found that GU extract supplementation increased the a* value and decreased the b* value and shear force of the breast muscle in broilers. Myoglobin in postmortem muscle can be oxidized to brown iron myoglobin during storage, resulting in a decreased a* value. GU and AM extracts enhanced antioxidant activity by reducing serum levels of malondialdehyde (MDA) and reactive oxygen species (ROS) in chickens (35), which could decelerate hemoglobin oxidation and thereby mitigate the decline in the a* value. The above results indicate that HGA can effectively enhance the production performance of broilers, including promoting growth and improving carcass traits and meat quality.

### 4.2 Immunity

The immune system affects animal growth efficiency, metabolic balance, and overall health by defending against pathogens and maintaining homeostasis, while the development of immune organs is crucial for immunity. The thymus is the center of cellular immunity and is the primary site of T cell development and maturation. The bursa of Fabricius functions as the primary site for B lymphocyte maturation, whereas the spleen orchestrates both cellular and humoral immunity in poultry (21). In this study, LGA significantly increased the bursa of Fabricius index, while HGA increased the thymus and bursa of Fabricius indices. Likewise, the relative weights of the bursa of Fabricius and thymus in quails linearly increased with supplementation of GU polysaccharide at 500–1,500 mg/kg (36). AM polysaccharides increased spleen weight and protected against heat stress-induced spleen damage in broilers (21).

Immunoglobulins (IgA, IgM, and IgG) serve as the primary effector molecules of the animal immune system, orchestrating a synergistic defense in humoral immunity through pathogen neutralization, toxin clearance, and immune response regulation. Our findings demonstrated that HGA enhanced the humoral immunity of broilers, as evidenced by elevated serum levels of IgA on day 28, IgM on day 56, and IgA, IgG, and IgM on day 84. This variation in immunoglobulin levels across different ages could be related to the development of the chicken immune system. In the early growth stage, the immune system is immature, and IgA exhibits a rapid response to environmental and dietary stimuli, whereas the production of IgG involves a slower process requiring B-cell maturation and the establishment of systemic humoral immunity (37). Cytokines have various functions, such as regulating immunity and participating in inflammatory responses. IL-2 mediates inflammation-driven immunity by enhancing T cell responses and promoting antibody secretion from B cells. IL-10 exerted anti-inflammatory effects through suppressing pro-inflammatory cytokines (e.g., IL-12, TNF-α, IL-1β, and IL-6), thus inhibiting excessive inflammatory responses and reducing cellular damage (38). TNF-α modulated diverse biological functions including inflammation, immunity, and lymphocyte homeostasis (39). We observed that HGA increased the serum levels of IL-10 and IL-2 at days 28 and 56, and IL-2 at day 84, while reducing TNF-α levels at days 56 and 84. Overall, HGA effectively promoted the development of immune organs and bolstered both humoral immunity and anti-inflammatory potential in broilers. This aligns with evidence that crude extracts of GU and AM improved the immune and antioxidant properties of chickens by decreasing the levels of TNF-α, IL-1β, and IL-6 (35). Certain active components of GU and AM have been demonstrated to enhance immune and anti-inflammatory responses. GU polysaccharides elevated serum IgA, IgM, and IgG concentrations and reduced IL-1β and IL-6 in broilers (40). Low-molecular-weight GU polysaccharides promoted IgM, IgG, and secretory IgA (sIgA) secretion (41). AM glycoproteins promoted the expressions of IL-2 in mouse splenocytes (42). Therefore, the enhancement of immune function in broilers by HGA is likely attributed to the combined effects of its diverse bioactive components.

### 4.3 Intestinal morphology and barrier

The structural integrity of intestinal villi, which constitute the primary sites for nutrient absorption, is critical for animal health. The increased villus height (VH) provides a larger absorptive surface area that is beneficial for nutrient absorption in broilers. Crypt depth (CD) correlates with enterocyte proliferative activity, where reduced CD values were accompanied by elevated cellular maturation and improved absorptive capacity (43). Our study revealed that HGA improved intestinal development and morphological structure, specifically during the early growth stages of broilers under normal conditions, as evidenced by increased duodenal, jejunal, and ileal VH and VH/CD and reduced CD at days 28 and 56.

The intestinal barrier facilitates the selective absorption of essential nutrients and immune sensing, while effectively restricting the entry of pathogenic molecules and bacteria. Tight junctions (TJs), primarily composed of integral membrane proteins (e.g., occludin and claudins) and cytoplasmic scaffolding proteins like ZO-1, regulate paracellular flux to prevent endotoxin, pathogen, and antigen penetration, maintaining tissue homeostasis (44). SIgA is secreted in the mucus layer as an immune-sensing and regulatory protein. This study found that HGA upregulated the expression of *OCLN*, *ZO-1*, and *CLDN1*, and elevated sIgA levels in the jejunal mucosa of broilers, demonstrating that HGA enhanced intestinal barrier function under normal physiological conditions. In support of these findings, previous studies have shown that GU and AM extracts attenuated diarrhea symptoms and oxidative stress induced by lipopolysaccharide (LPS), while increasing the levels of anti-inflammatory cytokines (35). Moreover, GU extract promoted the expression of *OCLN* and improved intestinal health (45), while GU polysaccharides increased VH and VH/CD and upregulated the expression of *OCLN*, *CLDN1*, and *MUC2* in broilers (40). In piglets, GU flavonoids improved intestinal architecture by increasing VH and VH/CD ratio (22).

### 4.4 Cecum microbiota

Intestinal microbiota serves as a protective microbial barrier, playing a pivotal role in modulating host metabolic, nutritional, and immune functions (46). Numerous studies have shown that both AM and GU improved gut microbiota balance and alleviated intestinal microflora disorders (47, 48). Our study revealed that HGA supplementation significantly enhanced cecal microbiota richness and diversity in broilers during the early growth stage and persistently modulated the microbial community composition across growth phases.

In this study, the cecal microbiota at the phylum level varied across different growth stages in broilers, but *Firmicutes* and *Bacteroidetes* were always predominant phyla, which was in line with previous study on broilers (25). *Firmicutes* participates in polysaccharide decomposition and contributes to the maintenance of intestinal homeostasis and health. An increased abundance of *Firmicutes* in the gut was positively correlated with feed efficiency in broilers (49). *Firmicutes* and *Bacteroidetes* jointly modulated host energy acquisition and metabolic processing. A higher F/B ratio was often associated with increased broiler body weight gain (50). *Desulfobacterota*, particularly the genus *Desulfovibrio*, suppressed the production of the gut hormone glucagon-like peptide 1 by producing H₂S, thereby affecting the host’s glucose metabolism. A reduction in *Desulfobacterota* correlated with improved gut barrier function (51). Dietary HGA supplementation increased the abundance of *Firmicutes* on days 28 and 56, resulting in a higher F/B ratio, while simultaneously reducing the abundance of *Desulfobacterota* on days 56 and 84, suggesting that HGA could enhance growth performance in broilers by reshaping gut microbiota composition.

We further investigated the changes at the genus level to determine which bacterial taxa drove HGA-induced restructuring of the cecal microbiota. Among the dominant bacterial genera, HGA increased *[Ruminococcus]_torques_group* at days 56 and 84, and *Rikenellaceae_RC9_gut_group* at day 84. *[Ruminococcus]_torques_group* has been shown to enhance intestinal barrier function by promoting short-chain fatty acid (SCFA) production, which impacts broiler growth and development (52). *Rikenellaceae_RC9_gut_group*, an SCFA-producing genus, effectively alleviated colitis induced by dextran sulfate sodium (53). Additionally, HGA reduced the abundance of *Bacteroides* on days 28 and 56. *Bacteroides* species are important commensal bacteria in the human and animal gut microbiota, but certain strains can become opportunistic pathogens under specific conditions.

Random Forest Classification analysis demonstrated that HGA altered the composition of cecal microbiota at the genus level in an age-dependent manner in broilers, thereby progressively remodeling the gut microbial structure. Notably, HGA increased the relative abundance of *unclassified_Lachnospiraceae* and *Negativibacillus* on day 28, *unclassified_Muribaculaceae*, *Enterococcus*, and *Butyricicoccus* on day 56, and *[Ruminococcus]_torques_group*, *Rikenellaceae_RC9_gut_group*, and *Enterococcus* on day 84. Studies have reported that members of the *Lachnospiraceae* family, including *unclassified_Lachnospiraceae* and the *Lachnospiraceae_NK4A136_group*, produced SCFA, maintain intestinal barrier integrity, and attenuate hyperinflammatory responses triggered by infection (54, 55). *Negativibacillus* abundance exhibited a positive correlation with body weight gain in mice (56), while dietary supplementation with *Enterococcus* enhanced laying hen performance (57). *Butyricicoccus*, a butyrate-producing bacterium, has been suggested to improve growth performance, suppress pathogen proliferation, and alleviate intestinal inflammation in broilers (58). *Muribaculaceae* degraded mucin monosaccharides, preserving the integrity of the mucus layer, and preventing pathogen invasion (59).

In contrast, HGA reduced the abundance of genera that have been reported to associate with inflammation and disease, such as *Campylobacter*, *Desulfovibrio*, and *Parasutterella*. *Parasutterella* and *Desulfovibrio* belong to *Proteobacteria*, a phylum that includes various pathogenic bacteria. *Parasutterella* contributed to chronic intestinal inflammation (60), and its increased abundance correlated with dysbiosis or reduced intestinal flora diversity (61). *Desulfovibrio* is a key producer of lipopolysaccharide (LPS), a potent inflammatory mediator. Qiao et al. (40) demonstrated that *Desulfovibrio* abundance was positively correlated with elevated serum levels of TNF-α, IL-1β, and IL-6, while GU polysaccharides reduced its colonization and suppressed LPS production. *Campylobacter*, a leading cause of acute bacterial enteritis, is primarily transmitted through poultry, especially broiler chickens (45). Although often asymptomatic in chickens, *Campylobacter* colonization led to subclinical enteritis and reduced performance (62).

Furthermore, ecological interaction analysis showed that HGA altered microbial interactions, forming a more interconnected network with a higher proportion of strong correlations, as evidenced by a greater average degree and denser microbial interactions. On day 28, HGA replaced *Faecalibacterium_prausnitzii* in the CON group with *Parabacterioids Johnsonii* as the core node, altering the correlation patterns. *Parabacterioides johnsonii* was a key butyrate-producing bacterium associated with anti-inflammatory and barrier functions (63). It was positively correlated with probiotics, including *unclassified_Oscillospiraceae*, which was involved in the nutritional metabolism of poultry (64), as well as *Intestinimonas_timonensis* and *Fournierella_massiliensis*, both of which enhanced immunity in broilers (65, 66). On day 56, the HGA group shifted *Mucispirillum schaedleri* to be the core node instead of *unclassified_Rikenellaceae_RC9_gut_group* in the CON group, which altered its correlation patterns. Additionally, both groups shared unclassified_*Rikenellaceae_RC9_gut_group* as a core node on day 84.

In summary, HGA modulated the gut microbiota toward a healthier profile by enriching taxa associated with host benefits, reducing potentially harmful taxa, and altering microbial interaction patterns. This restructuring could support metabolic efficiency, gut barrier function, and immune regulation.

### 4.5 Cecum metabolites

The intestinal microbiota establishes close dialogs with the host through its small-molecule metabolites, such as SCFA, amino acids, and bile acid derivatives, directly participating in the regulation of the host’s immune response, energy metabolism, and intestinal barrier function (67). These metabolites act as key mediators in the “dialog” between the gut microbiota and host, influencing many physiological processes. Our study revealed that HGA significantly altered the intestinal metabolic profiles through multiple pathways, including host-derived compounds, microbial metabolites, drug metabolites, and feed components, with the most pronounced effects observed during the early growth of broilers.

HGA upregulated carbohydrate digestion and absorption and the butanoate metabolism pathway by promoting butyric acid production, which was positively correlated with the increased abundance of SCFA-producing genera, such as *Ligilactobacillus*, *Synergistes*, *Marvinbryantia*, and *Furfurilactobacillus*. Studies showed that *Marvinbryantia*, a butyrate-producing bacterium, had anti-inflammatory effects (68) and helped in the regeneration of the intestinal mucosa (69). Butyric acid has been shown to mitigate intestinal injury by reducing inflammation and epithelial damage, restoring crypt structure, and enhancing barrier integrity through the upregulation of ZO-1, OCLN, and MUC-2 (70). At the molecular level, butyrate promoted fatty acid oxidation and boosted cellular energy production in intestinal cells (71). Additionally, it exerted anti-inflammatory effects by suppressing pro-inflammatory cytokines while simultaneously elevating IL-10 levels (72). As a G-protein-coupled receptor (GPCR) ligand, butyrate modulated immune responses by differentially regulating pro- and anti-inflammatory factors in epithelial and immune cells (73). Functioning as antibiotic alternatives, butyrate and its derivatives have been shown to augment growth performance, carcass quality, intestinal structure, and immune responses in broilers while alleviating intestinal inflammatory responses and oxidant stress (74). These results suggest that HGA could promote butyric acid production in the intestine, thereby enhancing the immune and antioxidant capacities, and intestinal barrier function of broilers.

Gut microbial metabolites also act as vital intermediaries in the communication between microbiota and the host. Bile acids (BAs) are synthesized in hepatocytes and produce primary bile acids (PBAs), such as cholic acid and chenodeoxycholic acid, which are generally conjugated with glycine or taurine (75). Most intestinal PBAs undergo enterohepatic recirculation to the liver; however, a minor fraction of PBAs reaches the hindgut for microbial deconjugation and metabolism into secondary bile acids that modulate host physiology. In this study, HGA enhanced PBA biosynthesis by increasing cecal levels of 3α, 7α, 12α-trihydroxy-5β-cholestanoate (THCA) and 3α, 7α, 12α-trihydroxy-5β-cholestan-26-al. These metabolites are critical intermediates in BA biosynthesis and gut microbiome interactions, with significant implications for host metabolism, immunity, and growth (76). THCA is an intermediate in the peroxisomal side-chain shortening of cholesterol to form bile acids like cholic acid, and disruption of THCA metabolism contributed to liver dysfunction (77). THCA and its derivatives are metabolized by microbial enzymes, such as bile salt hydrolases (BSHs) and 7α-dehydroxylase. Reduced levels of 7α-dehydroxylated BAs impaired gut barrier function and mucosal immunity, whereas secondary bile acids, such as deoxycholic acid (DCA), enhanced intestinal barrier integrity by promoting crypt regeneration and repair (78). Interestingly, our study also found THCA and 3α, 7α, 12α-trihydroxy-5β-cholestan-26-al were positively correlated with the abundance of bacteria involved in the degradation and metabolism of complex carbohydrates, including *Synergistes*, *Marvinbryantia*, *Holdemania*, *Furfurilactobacillus*, *[Ruminococcus]_torques_group*, *Lachnoclostridium*, and *Shuttleworthia.* Studies have demonstrated that an increased abundance of *[Ruminococcus]_torques_group* was significantly correlated with bile secretion and enrichment of the PBA biosynthesis pathway (79, 80). Multiple bacterial genera, including *Clostridium*, *Lactobacillus*, *Bifidobacterium*, and *Enterococcus*, exhibited BSH activity (81), and most lactic acid-producing bacteria possessed BSH enzymes (82). On the other hand, by leveraging their unique physiological functions, BAs selectively promoted or suppressed the growth of specific microbial populations, thereby altering gut microbiota composition (83). In short, HGA could regulate bile acid metabolism by influencing microbial composition, thereby improving the health and intestinal barrier function of broilers.

(3Z)-phycocyanobilin (PCB) was significantly upregulated in the cecum by HGA. PCB was a blue pigment that belonged to a class of tetrapyrrole compounds, demonstrating antioxidative and anti-inflammatory activities (84). Studies have found that PCB reduced inflammation by decreasing the levels of pro-inflammatory factors, such as IL-6 and IFN-γ (85). PCB also acted as aryl hydrocarbon receptor (AhR) agonists, upregulating heme oxygenase 1 (HO-1). HO-1 inhibited proinflammatory cytokine production in activated macrophages (86) while promoting IL-10 secretion (87). Moreover, PCB mimicked biliverdin to activate the anti-inflammatory signaling pathway through biliverdin reductase, thereby increasing IL-10 levels and exerting an anti-inflammatory effect (88). Correlation analysis showed that PCB was positively correlated with beneficial bacteria, such as *Holdemania*, *unclassified_Erysipelatoclostridiaceae*, *Parabacteroides*, *[Ruminococcus]_torques_group*, *Lachnoclostridium*, and *Shuttleworthia*. These results suggested that HGA could exert antioxidant and anti-inflammatory effects by promoting the accumulation of PCB.

Enoxolone (glycyrrhetinic acid), a metabolite of glycyrrhizin, exists in two configurations, 18α-glycyrrhetinic acid and 18β-glycyrrhetinic acid. It demonstrated a broad spectrum of biological activities, including antioxidant, anti-inflammatory, and antibacterial effects (89). The bioactive components of GU underwent biotransformation by the gut microbiota, generating derivatives (e.g., 18β-glycyrrhetinic acid) with enhanced pharmacological activity (90, 91). 18β-glycyrrhetinic acid exhibited notable inhibition of intracellular nitric oxide synthesis (92). Our study revealed that increased cecal enoxolone levels were significantly positively correlated with specific gut microbiota in the HGA group, including potential carbohydrate-degrading and SCFA-producing genera such as *Marvinbryantia*, *[Ruminococcus]_torques_group*, *Lachnoclostridium*, and *unclassified_Erysipelatoclostridiaceae* (93), as well as genera involved in bile acid and lactic acid metabolism, such as *Parabacteroides* and *Olsenella*. This suggests that intestinal probiotics could enhance the biotransformation of GA bioactive components directly or by modulating the gut microbiota environment. Additionally, enoxolone levels were negatively correlated with some potentially harmful bacteria, such as *Gallibacterium*, *Tuzzerella,* and *Prevotellaceae_Ga6A1_group*. These bacteria may disrupt the balance of the gut microbiome, trigger intestinal inflammation, and weaken intestinal barrier function. To sum up, HGA promoted the growth of probiotics, which, in turn, could enhance the biotransformation and utilization of bioactive components in GA, while also inhibiting the growth of harmful bacteria. This aligns with evidence that probiotics increased the baicalin bioactivity by facilitating its conversion into highly active compounds in the ileum, which in turn elevated the abundance of SCFA-producing bacteria in broilers (94).

## 5 Conclusion

In summary, supplementation with both 0.1 and 0.3% GA improved the growth performance of broilers, with the 0.3% GA (HGA) showing superior efficacy. HGA enhanced production performance by promoting growth and improving carcass traits and meat quality. Additionally, HGA bolstered immunity by facilitating immune organ development and modulating serum immune and inflammatory cytokine levels. It also improved gut health by promoting intestinal morphological development and strengthening the barrier function. Moreover, HGA reshaped the cecal microbiota and altered the cecal metabolome. These results suggest that 0.3% GA supplementation could be a promising candidate for a sustainable, antibiotic-free TCM-derived feed additive. However, as a combination containing multiple active complexes, the pharmacological mechanisms of its growth-promoting effects warrant further investigation.

## Data Availability

Cecal microbiome sequencing reads (16S rRNA) were deposited in the NCBI (https://www.ncbi.nlm.nih.gov/) under the accession number PRJNA1371690. Additional data related to this study may be requested from the authors.

## Footnotes

1
https://www.biocloud.net/

2
https://hmdb.ca/

3
https://metlin.scripps.edu

4
https://www.biocloud.net/

5
https://www.kegg.jp/kegg/pathway.html

6
http://metorigin.met-bioinformatics.cn/
